# Sensitivity of Remote App‐Based Assessment of Cognition to Sleep‐Mediated Differences in Cognitive Functioning Among Older Adults

**DOI:** 10.1002/alz.093482

**Published:** 2025-01-03

**Authors:** Rachel Rovere, Erin Liebenberg, Kimberly Halberstadter, David A Wolk, Dawn Mechanic‐Hamilton

**Affiliations:** ^1^ Penn Alzheimer’s Disease Research Center, University of Pennsylvania, Philadelphia, PA USA; ^2^ University of Pennsylvania, Philadelphia, PA USA; ^3^ Penn Alzheimer’s Disease Research Center, University of Pennsylvania, Philadelphia, PA USA

## Abstract

**Background:**

This study assesses the sensitivity of the mobile cognitive app performance platform (mCAPP), a mobile and engaging cognitive assessment tool, to participant sleep duration.

**Method:**

The mCAPP includes three gamified tasks: a memory task (“Concentration”), a stroop‐like task (“Brick Drop”), and a digit‐symbol coding‐like task (“Space Imposters”). For all games, shorter reaction times and fewer guesses indicates better performance. The cohort included 50 participants (72% female; age = 71.9±4.5; education = 16.8±2.3; 51% white; 49% Black/African American) without cognitive impairment who are enrolled in the Penn ADRC cohort. Sleep data was collected via actigraphy (Fitbit). Game performance and overnight sleep prior to gameplay were analyzed as a whole and grouped by fewer (quartile 1; Q1) and higher (quartile 4; Q4) minutes of sleep.

**Result:**

The average number of minutes of sleep was 378.0±96.5. Overall, minutes of sleep showed a significant negative correlation with reaction time on Space Imposters (ρ = 0.001). Participants with the fewest minutes of nighttime sleep performed slower than those with the most sleep on Space Imposters (Q1 M = 2.73; Q4 M = 2.40; p<.001), Brick Drop (Q1 M = 1.58; Q4 M = 1.31; p = .004), and Concentration (Q1 M = 5.25; Q4 M = 5.02; p = .006). There was not a significant difference in number of errors on Space Imposters (p = 0.53) or Brick Drop (p = 0.45) when comparing performance of participants with lower and higher quartiles of sleep, but there was a significant difference in the number of guesses on Concentration (Q1 M = 14.91; Q4 M = 12.80; p = 0.01).

**Conclusion:**

The mCAPP can remotely detect differences in cognitive performance related to nighttime sleep prior to gameplay. Future studies will examine differences in performance related to the time‐of‐day gameplay occurs (how long after overnight sleep) and whether performance is affected by daytime napping. A larger data set will also be used to fully determine the sensitivity of mCAPP to participant sleep duration and examine within‐participant effects.

